# Qualitative Analysis of Glass Microfragments Using the Combination of Laser-Induced Breakdown Spectroscopy and Refractive Index Data

**DOI:** 10.3390/s22083045

**Published:** 2022-04-15

**Authors:** Dávid Jenő Palásti, Judit Kopniczky, Tamás Vörös, Anikó Metzinger, Gábor Galbács

**Affiliations:** 1 Department of Inorganic and Analytical Chemistry, University of Szeged, Dóm Square 7, 6720 Szeged, Hungary; palastidavid@chem.u-szeged.hu; 2 Department of Optics and Quantum Electronics, University of Szeged, Dóm Square 9, 6720 Szeged, Hungary; jkopniczky@physx.u-szeged.hu; 3 Department of Physics and Chemistry, Hungarian Institute for Forensic Sciences, Mosonyi Street 9, 1087 Budapest, Hungary; vorost@nszkk.gov.hu; 4 Department of Applied and Nonlinear Optics, Institute for Solid State Physics and Optics, Wigner Research Centre for Physics, Konkoly-Thege M. Way 29-33, 1121 Budapest, Hungary

**Keywords:** glass samples, forensic analysis, laser-induced breakdown spectroscopy (LIBS), sample discrimination, chemometrics, multielemental sensing

## Abstract

We have successfully demonstrated that although there are significant analytical challenges involved in the qualitative discrimination analysis of sub-mm sized (microfragment) glass samples, the task can be solved with very good accuracy and reliability with the multivariate chemometric evaluation of laser-induced breakdown spectroscopy (LIBS) data or in combination with pre-screening based on refractive index (RI) data. In total, 127 glass samples of four types (fused silica, flint, borosilicate and soda–lime) were involved in the tests. Four multivariate chemometric data evaluation methods (linear discrimination analysis, quadratic discrimination analysis, classification tree and random forest) for LIBS data were evaluated with and without data compression (principal component analysis). Classification tree and random forest methods were found to give the most consistent and most accurate results, with classifications/identifications correct in 92 to 99% of the cases for soda–lime glasses. The developed methods can be used in forensic analysis.

## 1. Introduction

Glass is a common material; it is widely used in the industry, such as in the construction (e.g., windows, walls), automotive (e.g., windscreens, side windows, headlamps, mirrors), or optical and lighting industries (shades, lenses and other optical elements), as well as in households (e.g., tableware, containers, decorative elements) or in chemical and biological laboratories (e.g., volumetric glassware, test tubes) and other fields. From this variety of uses and prevalence comes the importance of glass as evidence in forensic investigations. Splinters and glass microfragments (<1 mm in size) often can be found at the locations of car accidents, car thefts, hit-and-run accidents, vandalism, burglaries, assaults, bomb attacks, etc. These microfragments are generated during the fracture of glass, can occasionally fly more than three meters away from the source and can be trapped in the clothing, shoes or hair of a person present at the scene; thus, the fragments can link people to each other or to locations [1,2,3]. Thus the instrumental analysis of glass samples is an important part of forensic investigations. There are two central questions in such investigations: (i) whether certain recovered glass fragments can originate from a given broken glass object (known sample) found at the scene (comparative analysis), and (ii) to what type of glass the recovered fragments may belong to (discrimination analysis). The exact identification of a glass piece is a very challenging task, given the huge variety of glasses present in an urban environment and also the compositional variations associated with the fabrication of glass products [1,2,3]. In fact, it is often more realistic to make exclusive than inclusive statements (e.g., assess which glass types/class a fragment can not be as opposed to stating what it can be). Due to this, the validity of the result of comparison/discrimination analysis of glasses is typically limited by the size of the library of samples (number of types/classes considered).

During forensic analysis, glass fragments are examined in several ways. Typically this includes refractive index (RI) measurements at standardized wavelengths, often with temperature programming, and the quantitative analysis of trace/minor elements by solid sampling instrumental methods with spatial resolution such as electron microscopy with energy dispersive spectroscopy (SEM-EDX) [4,5,6], micro X-ray fluorescence spectroscopy (µ-XRF) [7,8,9,10,11] and laser ablation inductively coupled plasma atomic emission (LA-ICP-AES) or mass spectrometry (LA-ICP-MS) [7,9,12,13], as well as particle-induced gamma ray emission (PIGE) and neutron activation analysis (NAA) [14]. Solution-based ICP-AES, ICP-MS or graphite furnace atomic absorption spectrometry are also used to obtain compositional information [15,16,17,18], but these approaches are time- and sample-consuming due to the need for grinding, melting and acid digestion of the glass sample, which cause significant dilution. If the fragment is not too small (e.g., it is at least several millimeters in size) then further characteristics, such as the color, thickness, luminescence, curvature and other surface features can also be assessed in addition to RI and elemental composition [2,3,19].

It has already been well established in the analytical literature that RI measurements compared to multielemental sensing of minor and/or trace elements have a much smaller efficiency in the discrimination of glasses. This is due to the fact RI values only depend very weakly on the minor/trace glass composition, and since most glass applications (e.g., other than optics) do not dictate the control of the RI, many glass types have similar refractive indices. At the same time, good results (85–99% accuracy) in glass discrimination or comparative analysis were obtained by using XRF, LA-ICP-MS or SEM-EDX trace elemental composition data alone or combined with RI data (e.g., [7,8,11,12]). Unfortunately, these techniques require bulky and costly instrumentation and have serious limitations in terms of the shape and size of the sample.

Laser-induced breakdown spectroscopy (LIBS) is a laser-based atomic emission spectroscopy technique. A short-duration, high-intensity laser beam is focused onto the surface of the solid sample, which evaporates the material of the sample located in the focal spot. The ablated material is also atomized and excited by the laser pulse, which leads to the formation of a microplasma. The analytical information about the elemental content of the sample is gained from the observation of the plasma emission. LIBS has a far more practical list of features that can be potentially well exploited for forensic analysis of samples, including glass samples. It is similar to LA-ICP-MS in terms of minimal sample preparation and high spatial resolution (micrometer-range), but does not use a complicated vacuum system (in contrast to both SEM-EDX and LA-ICP-MS). It is built around a robust, compact (even field-portable), reasonably cost-efficient and fast-to-use instrumentation and can work with almost any shape and size of solid samples and operate under ambient conditions. As opposed to XRF and SEM-EDX, it is sensitive to all elements, including the light or hard-to-ionize ones, which allows the recording of fingerprint-like, feature-rich spectra of the sample that contain a plethora of information about all major, minor and trace elements (down to the ppm level) [20,21,22]. Exploration of the applicability of LIBS for forensic analysis (e.g., for paper, ink, paint, etc. samples) in the literature was started more than a decade ago (e.g., [23,24,25]). In particular, forensic glass analysis by LIBS was started in 2005–2006 with the demonstrative publications of Almirall et al. [26] and Bridge et al. [12]. LIBS-based glass discrimination studies are still dominated by publications of Almirall and co-workers in the recent literature.

In a later study, Bridge et al. [13] examined four types of glasses (float glass from automobile windows, headlamps, side mirrors and brown beverage container glasses) which are commonly encountered in forensic cases. They used a combination of LIBS, RI and LA-ICP-MS data to improve the discrimination efficiency of the analysis. They found that combining RI and LA-ICP-MS has a higher discrimination efficiency than RI combined with LIBS. Glass is an optically transparent material, thus the laser wavelength is expected to have a significant influence on the ablation. Barnett et al. [27] examined the influence of the laser wavelength on automotive and NIST standard glasses and found that although 266 nm ablates more material, 532 nm produces higher electron density and more intensive emission. Later, the same group also compared the effect of 266 nm and 1064 nm laser wavelengths on the accuracy and precision of quantitative glass analysis, and it was established that the UV wavelength provides a better analytical performance [28]. Rodriguez-Celis et al. analyzed ten different car window (rear and side) glass samples using linear and rank correlation of LIBS data and obtained 91–98% accuracy in the identification. It was also reported that repetitive laser pulses on the same sample spot generally increase the analytical signal and decrease the relative standard deviation. This effect was attributed to the increased defects (cracks, roughening) generated by repeated ablation on the glass surface [29]. Naes et al. studied 41 automotive glass samples by several elemental microanalytical techniques, including LIBS [9]. Ten ratios were constructed from the combination of signal intensities measured for Al, Na, K, Ca, Fe and Sr spectral lines, which assisted in false exclusions and reducing false inclusions, which are important aspects of forensic examinations.

In their chemometric study, McIntee et al. suggested the adoption of a non-parametric permutation test for the discrimination of glass samples via their LIBS spectra, pointing out that any deviation from the normal distribution when the basis of similarity is the Pearson correlation coefficient [30]. El-Deftar et al. investigated Australian architectural window glass samples by LIBS, using pairwise comparisons based on a three-sigma rule, two-way ANOVA and Tukey’s HSD test in order to assess the discriminative power [31]. It was established that LIBS provides a fairly comparative efficiency (ca. 97%) in this application compared to LA-ICP-MS and µ-XRF. Gerhard et al. even demonstrated that the quantitative analysis of glass by LIBS can be performed with a better than 10% precision using the calibration-free approach that relies on plasma modeling [32].

Thus, the literature data so far suggest that LIBS has a good potential for forensic glass analysis, especially because it uses a cheaper, faster and easier examination technique. In our present study, we investigate the performance of LIBS for glass discrimination when the samples are microfragments (around or below 100 µm in size), where the homogeneity and laser ablation behavior of the samples are particularly important, because a large number of repetitive measurements are not possible. Our study focuses on the case of soda–lime glasses, because this represents the most widely used group of glass and therefore is also one of the most frequently studied types of forensic evidence. We paid particular attention to comparing and combining the discrimination performance of LIBS and RI. Because RI data are scalar numbers and LIBS spectra are large data vectors, their combination can not be efficiently done via data fusion (as opposed to, e.g., Raman and LIBS combinations). Instead, our approach was to use RI data for pre-screening.

## 2. Materials and Methods

### 2.1. Measurements

LIBS experiments were performed on a J-200 tandem LA/LIBS instrument (Applied Spectra, West Sacramento, CA, USA) equipped with a 266 nm, 6 ns Nd:YAG laser source and six-channel CCD spectrometer with a spectral resolution of around 0.07 nm at the Department of Inorganic and Analytical Chemistry, University of Szeged. LIBS spectra were recorded in the Axiom instrumentation data acquisition software using a 1 μs gate delay and 1 ms gate width, in the wavelength range of 190 to 1040 nm. The smallest spot size available on the instrument, 40 μm, was used throughout the experiments. The pulse energy was 15 mJ (with a 2% RSD) and the pulse repetition rate was 10 Hz. The ablation chamber was rinsed by a 99.995% purity argon gas flow at a flow rate of 0.5 L·min^−1^ (Messer Ltd., Budapest, Hungary), controlled by the mass flow controller of the LIBS instrument. Each sample was brought into focus by using the built-in dual (laser distance meter and camera driven) autofocus system of the J-200. If not indicated otherwise, 25 spectra of each glass sample were recorded (in 5 repetitions at 5 different locations).

Refractive index (RI) measurements were carried out at 589 nm by the oil immersion method using a GRIM 3 system (Foster + Freeman, Evesham, UK) at the Hungarian Institute for Forensic Sciences. All glass microfragments were mounted on separate microscope slides, covered with a few drops of silicone oil (Silicone oil B, Locke Scientific, Stalybridge, UK), crushed with a scalpel and covered with a thin glass cover plate. The fragments were observed under a phase-contrast microscope as the temperature of the sample was ramped at a rate of 4 °C·min^−1^. The RI value was determined using a temperature-matched calibration curve based on the measurement of 10 glass standard samples (Locke Scientific, Stalybridge, UK) measured on six different edges.

Laser ablation craters in the glass samples were investigated by using a Veeco Dektak 8 contact profilometer (Veeco, Plainview, NY, USA) available at the Department of Optics and Quantum Electronics, University of Szeged. The craters were fully mapped, not just line-scanned. The used stylus had a 2.5 μm radius of curvature and it was pressed against the sample by using 30 μN force. Recorded maps have a 330 nm lateral and 400 nm vertical stepping resolution.

### 2.2. Samples and Sample Preparation

Most glass samples investigated in the present study were provided by the Hungarian Institute for Forensic Sciences. Glass standards (Nos. 610, 612 and 614, National Institute of Standards and Technology, Gaithersburg, MD, USA) and some additional glass samples were supplied by the Department of Inorganic and Analytical Chemistry, University of Szeged. A total of 127 glass samples, representing four major glass types (fused silica, borosilicate, soda–lime and flint) were analyzed. Our study focuses on soda–lime glasses, hence the majority of the glass fragment samples (95 pieces) were of this type. Within the soda–lime group, float, container (beverage flask or jar), patterned and security (car side and rear windows, windshields) glasses were included. Since the majority of flat glasses are nowadays manufactured by the float method [33], float glasses represented the flat glass group in this study. The color of the glass fragment samples studied was varied; colorless glass, as well as tints of green, grey, brown, blue, yellow, and white, all occurred. We would like to stress though that the color of the samples was not given any special consideration during qualitative discrimination, especially because in the sub-mm size range almost all fragments are colorless. Table 1 provides an overview of the glass samples involved in the study. The type and sub-type classification indicated in Table 1 was used as a reference during the qualitative discrimination, and it was based not only on the known origin (use) of the given glass, but also on LIBS compositional analysis (see Section 3.1).

Our sample preparation followed the routine utilized by the Hungarian Institute for Forensic Sciences. Samples were typically splinters or macro (several mm) sized fragments which, after cleaning, were later crushed into smaller pieces for the purposes of the study. Surface contaminations were removed by spectroscopy-grade acetone, followed by, in case of macro fragments, wiping dry using Kimwipes tissues (Kimberly-Clark, Irving, TX, USA). Finally, glass fragments were attached to plastic sample holder discs with double-sided adhesive tape.

### 2.3. Data Evaluation

In this study, five well-established chemometric methods, namely linear discriminant analysis (LDA), quadratic discriminant analysis (QDA), classification tree (CT), random forest (RF) and principal component analysis (PCA) were tested. Details of these methods are described in chemometric textbooks, such in [34]; thus, here we only give a general overview.

PCA is one of the most commonly used data compression methods. It projects the measurements into a new space, where none of the variables are correlated, and aims to describe the data as best as possible. It is often used for the visualization of high-dimensional data and data preprocessing for other chemometric methods. LDA and QDA are commonly used for qualitative discrimination purposes. They are also considered data projection methods, but the projection is not used to describe the dataset, but to highlight the differences between existing groups. CT utilizes a so-called decision tree, whose construction consists of binary conditions (yes or no questions) and its purpose is to divide the data space along the existing variables into regions, and these regions are assigned to sample groups. The borders of the regions are determined in such a way as to minimize the misclassification rate. The results of this method are usually easy to interpret, because conditions can be directly assigned to physical parameters, such as wavelengths. The hierarchical tree structure, however, may make this method vulnerable to small changes in the data. Newer methods were developed to lessen this sensitivity and make CT more robust; one of the most successful of these is the random forest, which reduces the instability by repeatedly building decision trees, with the exclusion of randomly selected data points. The categorization of the unknown samples is decided by the majority vote of members of the forest [35].

Data processing was carried out using version 18.0 of the Clarity software (Applied Spectra, West Sacramento, CA, USA) and the open-source R Studio Desktop software package (v1.3). In the latter case, custom codes were developed using the Chemometrics, MASS, ALS, RPart and RandomForest packages.

## 3. Results and Discussion

There are some particular analytical challenges involved in the task of classification/discrimination of glass microparticles using LIBS-based sensing. One of these is that essentially the basis of discrimination is the elemental composition (even if spectral intensities are used), which is quite similar for most glass types, especially for soda–lime glasses [33]. The typical soda–lime glass composition contains ca. 70 m/m% SiO_2_ (silica), 13 m/m% Na_2_O (lime) and about 12 m/m% CaO (lime), plus one or more of up to fifty other compounds (primarily metal oxides or metal colloids) as minor components added to affect the color, viscosity, durability, heat absorption or some other physical property. A survey of the elements in these additives, such as B, Al, Mn, Co, Fe, Cr, Li, K, Cu, Pb, Sn, rare earth elements and others, can therefore help the investigator to narrow down the manufacturing method, and thus the qualitative analysis of the host glass item of the fragment in a crime scene (e.g., float glass, tempered glass, flint glass, container glass, etc.). The lack of elements, such as in fused silica, can also help the discrimination. Other elements, originating from the impurities of raw materials or from manufacturing tools, will also be present in glasses at trace (or minor) concentrations. These are characteristic of the particular batch or source of manufacturing and, therefore, their analysis is mainly useful for the comparison of individual glass fragments [19,33].

**Figure 1:**
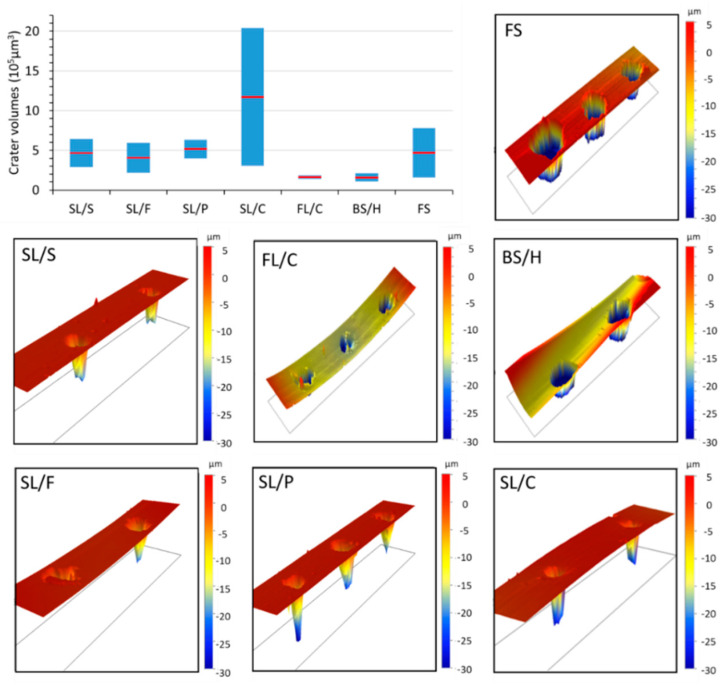
Contact profilometric data of the laser ablation craters on some selected glass types and sub-types. Ten laser pulses of 266 nm, with 40 µm spot size and 10 mJ pulse energy were delivered to the sample surface. The graph in the top left corner illustrates the large variation of the observed crater volume data for all glass types (the blue column represents the range and the red dot the mean value). The curvature of the profilometric maps is due to the actual curvature of the surface of glass fragments.

A further aspect concerning the challenges of basing the discrimination on concentrations arises from the fact that there is a significant difference between the nanosecond laser ablation behavior of glass types. As is illustrated in Figure 1, contact profilometric analysis of the ablation craters reveals that the shape, but more importantly the volume, of the craters (and thus the ablated mass) largely varies across glass types and sub-types. For most glass (sub-)types, such as fused silica and soda–lime glasses, the relative variation in ablation crater volumes is as large as 50% or more. Mostly, crater depth differences account for the variation in crater volumes, but please also note the crater rims, which often have jagged and protruding edges. This translates into concentration inaccuracies unless matrix-matched calibration or some form of signal normalization is applied to all calibration standards and samples. Unfortunately, none of the signal normalization approaches (e.g., ablation crater volume, acoustic signal, plasma temperature, internal standardization, etc.) generally proposed in the LIBS literature [36,37] are very practical or directly applicable to the analysis of a variety of glass microfragments. The use of matrix-matched calibration is hindered by the lack of a priori glass type information in a forensic glass discrimination study. Thus, in the present study, we decided to directly use the raw LIBS spectra (with data compression if needed) as an input dataset for qualitative discrimination purposes.

It is easy to see that for a reliable qualitative analysis of microfragments, it is imperative that the glass composition is homogeneous. If it is not the case, then the subtle differences in composition can be effectively buried in lateral or depth variations in the (trace) elemental composition. Luckily, an experimental assessment of the dissimilarities in composition can be performed with LIBS on sub-millimeter glass fragments, as LIBS analysis has a spatial and depth resolution in the micrometer range [21]. We investigated the intra-fragment homogeneity of our glass samples by collecting LIBS spectra from five laser pulses delivered at five locations within each glass fragment. Spectra within each fragment across locations were then compared to the mean spectrum using the linear correlation function, where a Pearson correlation coefficient value of 1 and 0 indicate full similarity and full dissimilarity, respectively (assuming positive intensities) [38]. We found this measure of spectral similarity very effective for several other sample types in our LIBS studies (e.g., [24,25,39,40]).

**Figure 2:**
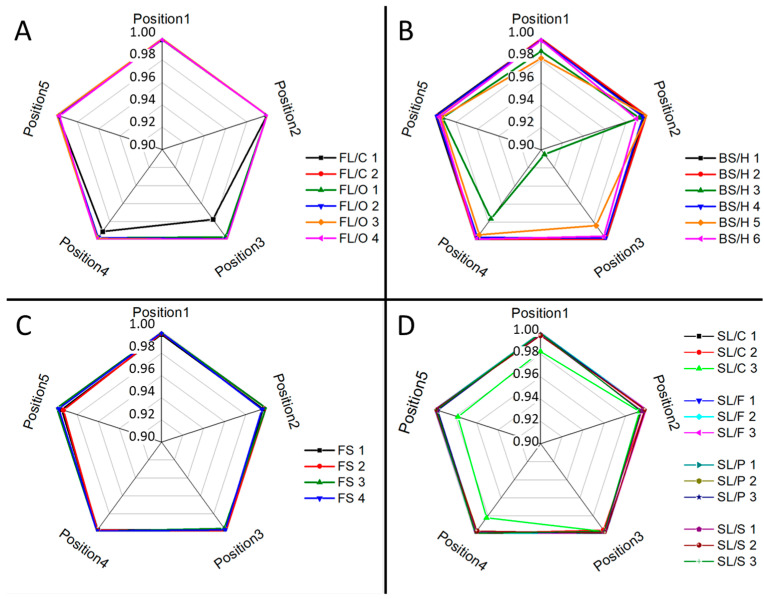
Intra-fragment lateral compositional variations for different glass types: (**A**) flint; (**B**) borosilicate; (**C**) fused silica; (**D**) soda–lime as assessed by comparing LIBS spectra taken at various locations by the linear correlation function. The average spectrum across the depth was taken as reference during the comparisons. The plots show the correlation coefficient on their radial axes.

**Figure 3:**
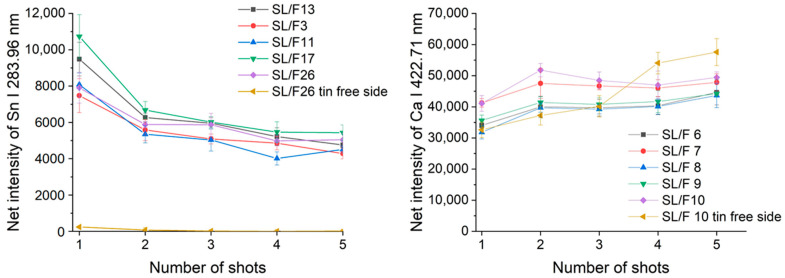
Depth-related compositional variations for float glass fragments, as assessed by plotting the net LIBS intensity of spectral lines of two selected elements as a function of the number of laser shots delivered. Error bars indicate scatter across intra-fragment locations at the same depth.

Ray plots in Figure 2 show the observed lateral intra-fragment spectral variations for four glass types. Figure 3 illustrates the change in the spectral line intensity of some elements as a function of depth (laser shots). In this latter investigation, we only included some of the float glass samples, which allows an easy demonstration of the effect of the manufacturing process on the surface trace element concentrations.

As expected, it was found that there is a reasonable similarity in the location-related LIBS spectra (correlation factors are at least 0.98, with only one borosilicate glass exception), which indicates a generally good lateral homogeneity. Depth-resolved comparisons reveal that elemental depth profiles are also typically flat, except for Sn, which shows a several times higher concentration in the top layer, which gradually decreases with the depth. This is the result of the float glass manufacturing process, which actually uses a bath of molten Sn (a liquid with only 505 K melting point, and a high density of around 7 g/cm^3^) for floating a sheet of glass (a density of 2–3 g/cm^3^) to make it smooth and flat—during this process, Sn diffuses into the glass [33]. This variability of Sn LIBS intensities also suggests that discrimination analysis of glass fragments (of a potentially float glass sample) should either start with screening the spectra for Sn and either use a surface of the glass shard which does not have significant Sn content or exclude (mask out) all Sn lines. The former approach was followed here. Since the lateral and depth-related concentration variance was neglectable, in further discussion we treated the spectra individually.

### 3.1. Glass Type Classification

**Figure 4:**
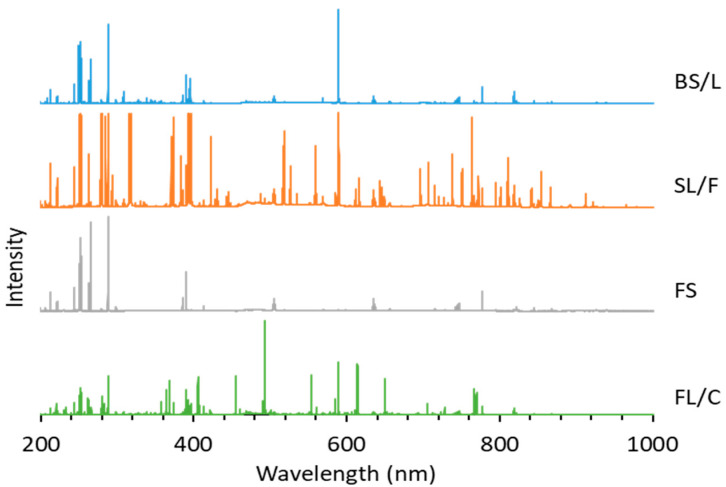
Representative LIBS spectra of borosilicate, soda–lime, fused silica and flint glass samples. The intensity scale of each spectrum is normalized to the highest peak intensity.

**Figure 5:**
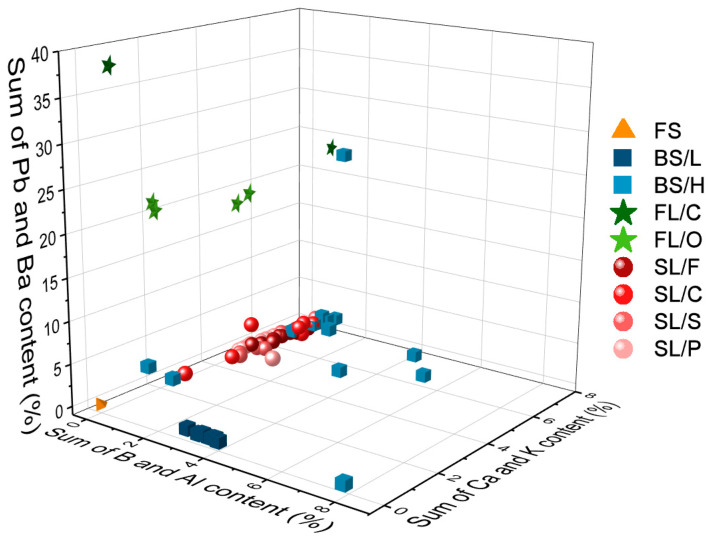
Classification of glass types according to the pairwise aggregated concentration of some indicator elements (Pb + Ba, B + Al, Ca + K). Quantitative analysis was based on calibration using the NIST 61X glass standard series. Markers of different glass types have different colors, while sub-type markers differ in shape.

**Figure 6:**
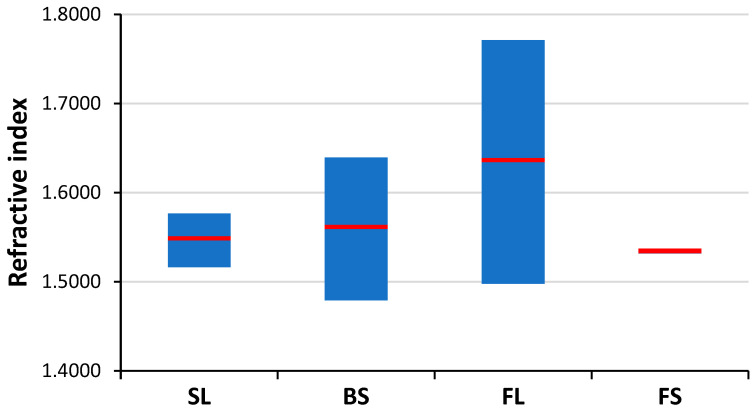
The spread (min, mean, max) of refractive index data of the studied glass types [41].

The possibilities, but also the challenges, of using LIBS analysis for the classification of distinct glass types based on their major and minor elemental composition are illustrated by representative spectra of glass types shown in Figure 4. It is easy to notice that the spectra belonging to different types have significant differences. As expected, the spectrum of fused silica is the simplest and contains only Si and O lines. In all other cases, the spectrum reflects the presence of a significant amount of B and Al (in borosilicate glass), Na, K and Ca (in soda–lime glass) and Ba and Pb in the flint glass. These elements are known to be used in the production of glass as additives. The pairwise aggregated concentrations of the chosen six indicator elements (Pb, Ba, B, Al, Ca, K) shown in Figure 5 allow the clear discrimination of several glass types, such as fused silica (low concentration of all elements), flint glass (high Pb + Ba content), soda–lime glass (low Pb + Ba but high Ca + K content) or borosilicate glass (low Pb + Ba as well as Ca + K, but high B + Al content). Quantitative evaluation was carried out using the Ca I 422.7 nm, B I 249.7 nm, Pb I 405.8 nm, K I 766.5 nm, Al I 309.2 nm and Ba II 455.4 nm spectral lines, which had no notable spectral interference. It may also be added that float glasses can also be easily separated from other soda–lime types based on the presence of a significant surface Sn content. However, while, e.g., data points for all soda–lime glasses cluster independently from the manufacturing process/intended use, automotive headlight shield glasses, which are generally expected to have similar toughness and low thermal expansion coefficient to borosilicate glasses, exhibit greatly varying formulation. Headlight glasses not only have additive concentrations distinctly different from borosilicate glasses, but their composition widely scatters within their own group. In spite of these conditions, LIBS—even in this rudimentary approach—still has a much greater discrimination potential for glass types than RI data (see Figure 6) or density [33].

### 3.2. Soda–Lime Glass Sub-Type Classification Using LIBS Data

**Figure 7:**
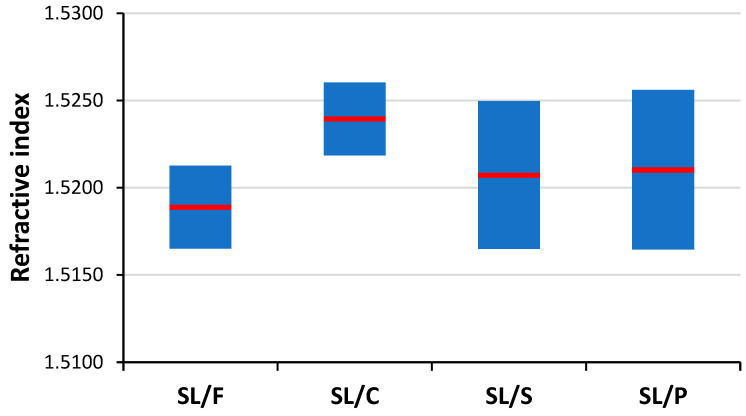
The experimental spread (min, mean, max) of refractive index data of the studied glass sub-types.

Soda–lime is by far the most common glass type, hence it is also often presented in forensic investigations as evidence. Consequently, the task of discriminating the soda–lime glass sub-types based on the analysis of microfragments is one of great practical importance. As it can be seen in Figure 7, refractive index data are weak indicators of soda–lime glass sub-type glasses, as there are significant overlaps between the RI data. LIBS data, if used directly as a source of elemental composition data, are also not reliable means of discrimination between these glass sub-types (see Figure 1 and Section 3.1). However, chemometric evaluation of LIBS data arrays has been proven very effective in the discrimination of other sample types [21], thus we put some of them to the test here as well.

**Figure 8:**
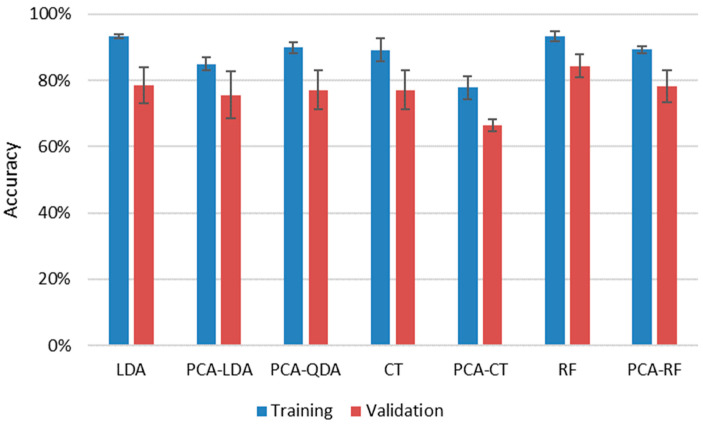
Cumulative (weighted mean) classification accuracy of various data evaluation methods for the assessment of the four sub-types of soda–lime glass samples by using LIBS data. Error bars are based on three replicate analyses using randomized separation of sample spectra to training and validation sets.

LDA, QDA, CT and RF methods were employed both with and without dimension reduction of data by PCA. Due to limitations in the number of variables for QDA (it cannot handle more variables than the number of objects belonging to the smallest group), we could not test QDA directly, only with PCA. During data reduction, the number of principal components was optimized by scrutinizing the elbow point of scree plots. In the application of all methods, LIBS spectra of samples were separated randomly into training and validation (testing) sets, keeping the ratio of the number of spectra to around 7:3. Three repeated evaluations were carried out, during which the samples were randomly redistributed into training and validation sets. Since the LIBS data of all samples showed good repeatability (Section 3.1), neither normalization nor scaling was performed prior to data analysis. The results of all approaches are summarized in Figure 8.

It is immediately apparent that all four methods provided decent cumulative accuracies in training, between 93% and 89%, and the repeatability of the classification was also very good at only a couple of percent. The reliability of the results (closeness of results for training and validation data sets) was also good, reflected by the closeness of accuracy values obtained for validation sets to those of training sets. Data compression was clearly found to be disadvantageous, as it decreased the accuracy by 5% to 10% for the three methods, for which results with uncompressed data could also be processed. This is probably due to the loss of information occurring during dimensionality reduction. The best, and actually identical, results were given by uncompressed RF and LDA methods (93.3%) for the training sets, but if the average of the results for a given method with training and validation data sets are considered, then RF slightly outperformed LDA (88.8% as opposed to 85.9%). The success of RF is not surprising as it is known to handle high dimensionality data very efficiently and is considered to be one of the highest accuracy classification methods. RF data evaluation of LIBS data has been found to be very efficient in the literature recently for a variety of sample types, from biological samples to ceramics and alloys to rocks [39,40,42,43].

In the confusion matrices presented in Table 2 one can notice that the security, patterned and container glasses each get confused for float glass the most. This suggests that the spectra of float glasses are lacking characteristic features, which might be explained by the lack of any special additives such as colorants. With the exception of security glasses, all other subtypes produce significantly lower accuracies for the validation sets. It is probably due to the more strict regulations applied to security glasses.

The random forest method provides the user with information about the “relative importance” of every variable (based on the frequency of their occurrence and success rate in tree conditions). Since in our case the variables are atomic emission wavelengths and they are linked to elements, these importance values indicate how much the model relies on the intensities of certain spectral lines. High importance wavelengths here could be assigned to metals present in glass additives (e.g., color modification or altering some other characteristics), such as 455.40 nm for Ba, 841.75 nm for K, 267.71 nm for Cr, 261.19 for Fe, 234.78 nm for Co and Fe, and 339.10 nm for Ni.

### 3.3. Identification of Individual Soda–Lime Glass Samples Using Classification Methods on a Combination of LIBS and RI Data

The most information has to be mined from the experimental data during the qualitative analysis of individual glass samples, when an unknown sample is compared to a database of known samples. This situation is common and clearly highly important during the forensic examination of evidence, when examiners assess the probability of a fragment potentially originating from a known glass sample from the scene (comparative analysis or pairwise comparisons)—or more commonly, how certainly it can be stated that two samples are not identical. The reason that the question is phrased like this is that in the case of such mass-produced and quite homogeneous samples such as glasses, the chance is higher that two samples are falsely identified as coming from the same sources (“identical”). Present practices in forensic examinations handle this analytical task often by refractive index comparisons. Although the precision of RI data of glass samples is very good (assured by four decimal digit measurements and the homogeneity of glass samples), we found that the reliability of such pairwise comparisons based solely on RI data is not sufficient. Only a small portion (4–32%) of samples belonging to the same soda–lime sub-type showed significantly different RI values.

**Figure 9:**
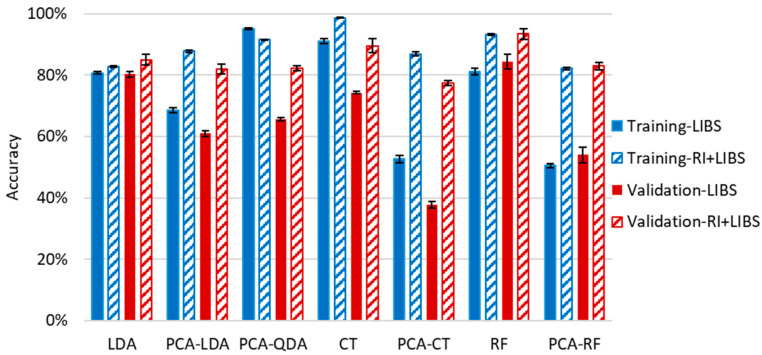
Cumulative (weighted mean) classification accuracy of various data evaluation methods for the identification of 100 individual soda–lime glass samples by using only the LIBS data and combining it with RI pre-screeing. Error bars are based on three replicate analyses using randomized separation of sample spectra to training and validation sets.

Because the glass sub-type classification proved to be quite accurate in Section 3.2, we intended to test whether the chemometric classification methods could also be efficiently employed if one expands the number of classes to the actual number of samples. In other words, we intended to assess the accuracy of individual soda–lime glass sample identification using classification methods. It has to be added, though, that this approach assumes that the real source glass object is included in the known sample set, as there is no “no match” result. To test this “identification” approach, and along the concepts outlined in the previous section for soda–lime sub-types, we performed repeated tests for the application of LDA, QDA, CT and RF methods. Since in the previous section we proved the possibility of the sub-type identification, in this section we focus on the classification of individual samples within an already identified sub-type. The results, which can be seen in Figure 9, indicate that LIBS data alone with any of these data evaluation methods do not provide sufficient discrimination power and reliability. Although in some cases the accuracy reaches 90% (CT and PCA-QDA), typically it is between 50% and 80%. Great differences in the accuracies for the training and validation sets can also be observed, with the only exceptions of RF and LDA. The trend that the evaluation of PCA-compressed data sets performs significantly poorer than that of uncompressed ones is the same as found in the previous section for glass sub-type classification.

**Figure 10:**
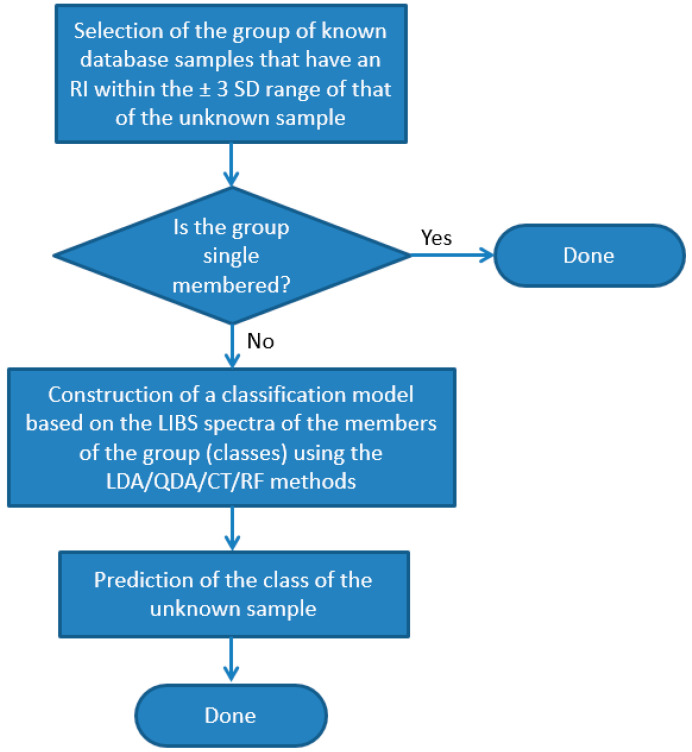
Flowchart of the process of identification (classification) of individual glass samples using the combined RI and LIBS approach.

In order to improve the accuracy of the identification of individual samples, we also carried out the test using combined RI and LIBS datasets. In the first step of this approach, we run a comparison of the RI data of the unknown sample against the RI data of all other soda–lime glass samples in the same sub-type. All RI data was represented in this test with a mean and a range set by ±3 times the standard deviation based on six repeated RI value determinations. Two samples were considered to not have significantly different RI values if the range of their RI values overlapped with each other. If the match was unique, then the identification was considered terminal and successful. Far more commonly, however, the match was not unique but involved several database samples (close hits). In these cases, as the second stage of identification, a chemometric evaluation of the LIBS data was carried out, but it only included those samples of the database (known samples) which were found not distinguishable by the RI values (Figure 10). We executed this comparative procedure for all 95 soda–lime glass samples, but as noted above, the sub-types were treated separately. The results given in Figure 9 are the ratios of all properly classified spectra to all investigated spectra.

The performance of this approach is consistently very good; the accuracy is around or above 80% for all chemometric methods. The reliability and repeatability of the results are also excellent. Again, CT and RF methods gave the highest overall accuracies of 95.0% and 93.5%, respectively. It is a considerable improvement considering the ca. 20% and 80% accuracy of the RI and RF, respectively, when used alone. The average number of close RI hits was found to be around 3.5, but this number depends highly on sample quality. For example, the average number of close RI hits for SL/P glasses was found to be 2.5, while it was 4.2 for SL/F glasses. The highest number of close RI hits was found among the SL/F samples as well, where some of the samples were found to have an RI value that was nearly identical to that of eight other samples.

## 4. Conclusions

We have successfully demonstrated that although there are quite a few analytical challenges involved in the forensic examination-oriented qualitative discrimination analysis of glass microfragment samples, the task can be solved with very good accuracy (90%+) and reliability with the multivariate chemometric evaluation of LIBS data, or alternatively in combination with a pre-screening based on RI data. The RI pre-screeing substantially raised the accuracy of the classification. On average, the improvement was 15%, but for certain methods, it reached 40%. The qualitative analysis can be successful even in the case of very small differences in glass quality, such as between the sub-types of common soda–lime glasses. Classification tree and random forest methods were found to give the most consistent, most accurate results. In several aspects, our study and the findings are more detailed than former studies on the qualitative LIBS analysis of glasses in the literature. These novel and improved aspects include the fact that we investigated far more samples (127) than any other studies, and uniquely focused on sub-mm sized glass fragments. We not only discussed the performance in glass type classification, but also the identification of individual glass samples by using classification methods. Apart from LIBS and RI data, the laser ablation behavior of different glass types and sub-types were also studied and described, including ablation crater volumes and laterally and depth-resolved element distribution.

## Figures and Tables

**Table 1:** An overview of the glass samples involved in this study.

Type	Sub-Type	Identifier	Number of Samples
fused silica	optical glass	FS	5
flint	crystal glass	FL/C	2
flint	optical glass	FL/O	4
borosilicate	headlight shield	BS/H	13
borosilicate	laboratory glass	BS/L	7
soda–lime	float (flat)	SL/F	23
soda–lime	container	SL/C	25
soda–lime	patterned	SL/P	22
soda–lime	security	SL/S	25

**Table 2:** Confusion matrices for LDA and RF classification used on soda–lime glass sub-types based on LIBS data.

LDA Training		True Class				LDA Validation		True Class			
		SL/S	SL/P	SL/C	SL/F			SL/S	SL/P	SL/C	SL/F
**Predicted class**	**SL/S**	90.9%	1.2%	0.2%	1.3%	**Predicted class**	**SL/S**	90.8%	3.0%	0.0%	4.8%
	**SL/P**	1.8%	94.2%	0.7%	4.3%		**SL/P**	1.0%	71.0%	8.0%	13.3%
	**SL/C**	0.4%	0.4%	95.7%	2.0%		**SL/C**	0.2%	0.2%	74.2%	5.0%
	**SL/F**	6.9%	4.1%	3.5%	92.5%		**SL/F**	8.0%	25.7%	17.8%	77.0%
**RF Training**		**True Class**				**RF Validation**		**True Class**			
		**SL/S**	**SL/P**	**SL/C**	**SL/F**			**SL/S**	**SL/P**	**SL/C**	**SL/F**
**Predicted class**	**SL/S**	92.4%	2.1%	0.0%	2.0%	**Predicted class**	**SL/S**	98.0%	5.0%	0.0%	2.9%
	**SL/P**	0.1%	93.2%	1.6%	3.3%		**SL/P**	1.0%	85.5%	11.2%	7.8%
	**SL/C**	0.0%	2.2%	96.1%	3.3%		**SL/C**	0.0%	0.6%	73.7%	9.5%
	**SL/F**	7.5%	2.4%	2.3%	91.5%		**SL/F**	1.0%	9.6%	15.2%	79.8%

## Data Availability

Not applicable.
